# Evaluation of Residual Toxic Substances in the Stomach Using Upper Gastrointestinal Endoscopy for Management of Patients With Oral Drug Overdose on Admission: A Prospective, Observational Study

**DOI:** 10.1097/MD.0000000000000463

**Published:** 2015-01-30

**Authors:** Masato Miyauchi, Makiko Hayashida, Hiroyuki Yokota

**Affiliations:** From the Department of Emergency and Critical Care Medicine (MM, HY); and Department of Legal Medicine (MH), Nippon Medical School, Tokyo, Japan.

## Abstract

The guidelines on the indications for gastric lavage were published in 1997, and a less-aggressive initial approach has been used for poisoned patients. Clinical studies have shown that the outcomes of retrieval of residual toxic substances in the stomach are variable and that no beneficial effect is obtained. However, the presence of residual toxic substances in the stomach before gastric lavage has not been estimated. The objective of this study was to evaluate the residual stomach contents on admission of patients with oral drug overdoses using upper gastrointestinal endoscopy.

A 2-year prospective study of 167 patients with oral drug overdoses was performed. Endoscopy was performed on admission to observe the gastric body, fornix, and pyloric antrum. Patients were classified into 3 groups according to the digestive phase (tablet/food phase, soluble/fluid phase, and reticular/empty phase). The groups were compared with respect to time elapsed since ingestion, and numbers and variety of orally overdosed drugs.

The numbers of patients in each phase were as follows: tablet/food phase, 73; soluble/fluid phase, 50; and reticular/empty phase, 44. The tablet/food and soluble/fluid phase groups contained the greatest numbers of patients who presented within 1 to 2 hours since ingestion. In the tablet/food group, only 12 of 73 patients (16%) presented within 1 hour since ingestion, and 3 patients presented >12 hours since ingestion. In the soluble/fluid phase group, only 9 of 50 patients (18%) presented within 1 hour since ingestion, and 2 patients presented >12 hours since ingestion. The reticular/empty phase group contained the greatest number of patients presenting within 2 to 4 hours since ingestion, and 3 patients presented within 1 hour since ingestion. The residual stomach contents before lavage were variable in all of the groups.

The residual gastric content before the performance of gastric lavage is variable in overdosed patients on admission. This may influence the efficiency of gastric lavage with respect to retrieval of residual toxic substances in the stomach. This study may contribute to the development of a strategy for treating patients who have orally overdosed on drugs in the future.

## INTRODUCTION

The numbers of both drug overdoses and suicides are increasing in Japan,^1^ making treatment of patients with drug overdoses an urgent problem. The first guideline for managing intoxicated patients was published in 1997.^2^ A less-aggressive approach during initial treatment was deemed appropriate after the guidelines on the indications for gastric lavage were published in 2004.^3^ These guidelines stated that there was no role for routine use of gastric lavage. However, whether a patient in the emergency department has overdosed is not always known. Furthermore, the status of residual toxic substances in the stomach is unclear in drug overdose situations. When a patient has overdosed on high-risk drugs, including tricyclic antidepressants,^4^ clinicians are faced with the dilemma of whether the drug contents in the stomach should be retrieved. Both guidelines stated that there was a lack of evidence regarding benefits, and that lavage should not be routinely performed in poisoned patients. However, these conclusions were drawn from animal studies and experimental studies of volunteers. In clinical studies of poisoned patients, various treatments have been evaluated, the outcomes of retrieval of residual toxic substances from the stomach were variable, and no beneficial effects were found.^5^ However, the patient's phase of gastric emptying before the performance of gastric lavage is often unknown. The residual contents of the stomach are not usually examined in detail. In this study, we evaluated the residual toxic substances in the stomach using upper gastrointestinal endoscopy on admission of patients with drug overdoses. Such examination may adequately reveal the residual content before gastric lavage. These results may help to establish firm indications for gastric lavage and provide guidelines for its use in patients with oral drug overdoses.

## METHODS

This study was approved by the Ethical Committee, Nippon Medical School, Tokyo, Japan, for research purposes.

### Setting and Data Collection

This prospective study was performed during a 2-year period. The inclusion criterion was presentation to our advanced emergency department for an oral drug overdose. The exclusion criteria were as follows: gastric lavage had been administered prior to arrival, the number of oral drugs that had been taken and/or the time that had elapsed since ingestion was unknown, and only a liquid medication had been ingested.

Informed consent was obtained after the details regarding the study objective and contraindications for endoscopy had been fully explained to the patients or the patients’ relatives if the patients exhibited consciousness disturbance. Confirmation of overdose was obtained by taking a careful history from the patient or an accompanying family member, or by inspecting the container labels and residual tablets or capsules. The patients received routine emergency treatment. After respiratory and circulatory stabilization, endoscopy (XQ 260; Olympus, Tokyo, Japan) was performed by emergency physicians. The patients were placed in the left lateral decubitus position, and the endoscope was slowly advanced. The residual contents of the stomach were observed. Demographic data (age and sex), the time of ingestion, and endoscopy results on admission were recorded for each patient. If the patient had a disturbance of consciousness, the timing and number of ingested drugs were determined after awakening.

### Data Processing

The residual contents of the gastric body, fornix, and pyloric antrum were estimated. We believed that the form of gastric contents was an important indicator of digestive processes and time elapsed since from ingestion.^6^ We therefore classified the endoscopy findings into the following 2 sets of 3 groups: tablet/food phase, soluble/fluid phase, and reticular/empty phase according to stage of the digestive process^7^; and small, moderate, and large according to the amount of drugs assessed by endoscopy as being in the gastric contents.^8^ In the tablet/food phase, residual tablets and/or food remained and were formed and recognizable in solid form. In the soluble/fluid phase, the tablets were digested and the stomach contents were in solution. In the reticular/empty phase, the residual substances had adhered to the stomach wall at the end of digestion or were not seen in the stomach. Data were recorded using printed forms and later transferred to a Microsoft Excel 2010 worksheet (Microsoft Corp, Redmond, WA).

### Statistical Analysis

Statistical assessment of the collected results was carried out by computerized multivariate analysis using Microsoft Excel 2012. The results are presented as mean ± standard deviation (continuous variables) or percentages (categorical variables). Student *t* test (for continuous variables) was used to compare clinical characteristics wherever appropriate. Statistical significance was assigned to comparisons with *P* values of <0.05.

## RESULTS

**FIGURE 1:**
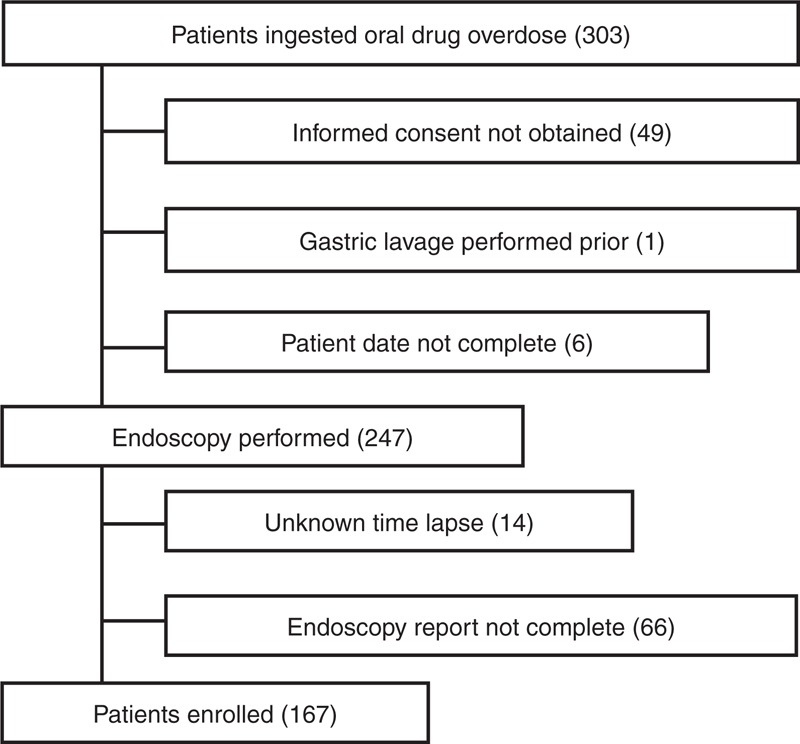
Flow chart of patient enrolment.

Of 303 patients seen for oral drug overdoses, 167 patients were enrolled in the study (Figure 1). The remaining 136 patients were excluded because informed consent was not obtained (n = 49), gastric lavage had been performed prior to arrival (n = 1), the patients’ data were not complete (n = 6), the time that had elapsed from ingestion to treatment could not be accurately determined (n = 14), or endoscopy reports in the stomach were not complete (data regarding the gastric body, fornix, and/or pyloric antrum were absent) (n = 66).

All patients were evaluated on admission. In total, 139 (83%) were male, and the mean patient age was 32.1 ± 10.3 (range, 15–71) years. No patients exhibited contraindications for endoscopy, such as aspiration, hypoxia, or oropharyngeal or gastric trauma.

**FIGURE 2:**
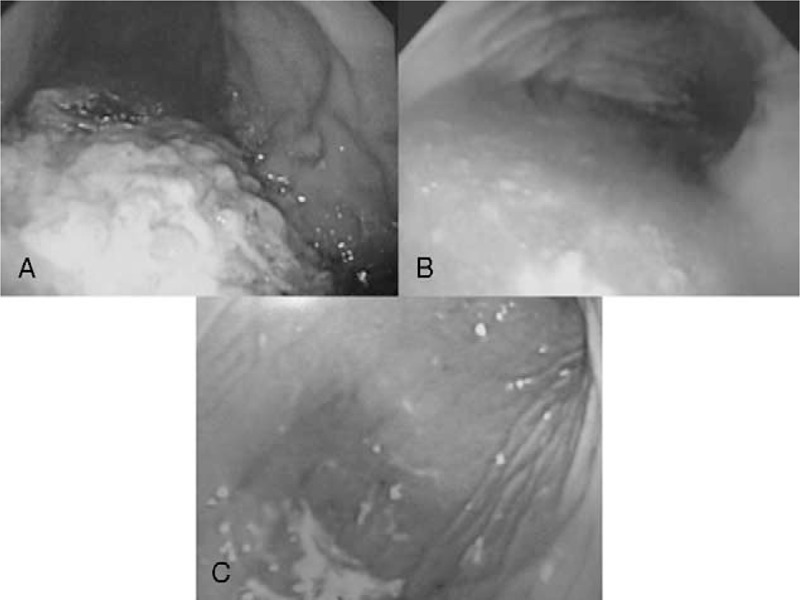
Typical photographs showing stomach during the 3 phases of digestion: tablet/food phase (A), soluble/fluid phase (B), and reticular/empty phase (C).

Figure 2 shows typical photographs of the 3 phases of digestion. The numbers of patients in each phase were as follows: tablet/food phase, 73; soluble/fluid phase, 50; and reticular/empty phase, 44 (Table 1). There were no correlations between the phase of digestion and age or sex among the 3 groups. However, the time since ingestion was significantly shorter in the tablet/food phase group (*P* = 0.003) and the soluble/fluid phase group (*P* = 0.013) than in the reticular/empty phase group. Similarly, there were significant associations between the amounts of ingested drugs and the tablet/food and soluble/fluid phases of digestion compared with the reticular/empty phase.

**TABLE 1:**
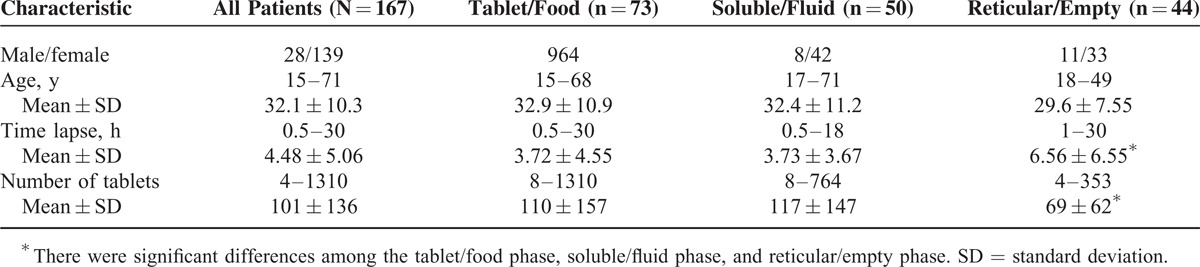
Clinical Characteristics of All Patients According to Digestive Phase During Endoscopy

**FIGURE 3:**
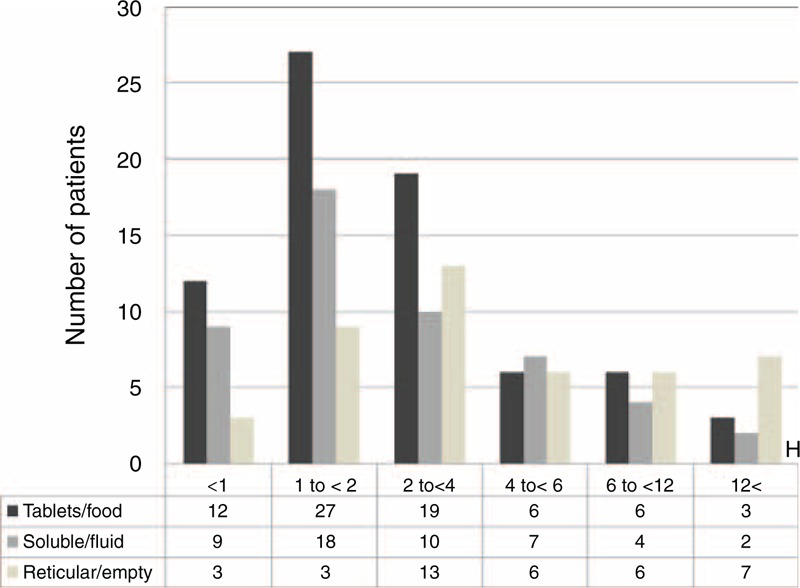
Correlations between time elapsed and numbers of patients.

**FIGURE 4:**
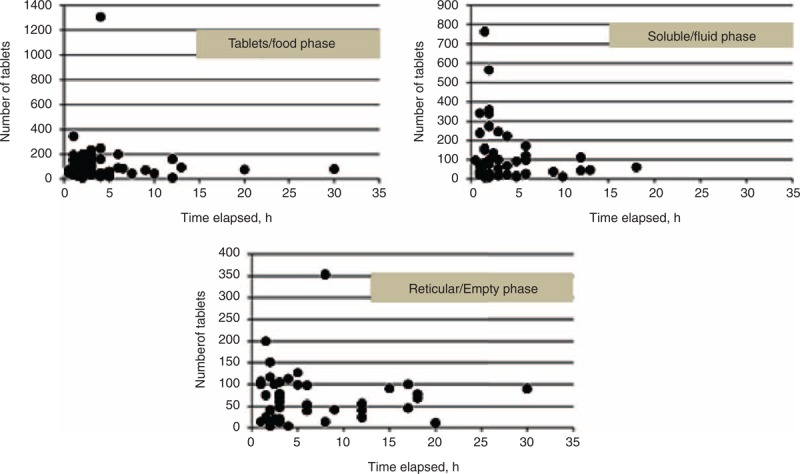
Correlations between time elapsed and numbers of tablets ingested.

Figure 3 shows that the tablet/food and soluble/fluid phase groups contained the greatest numbers of patients who presented within 1 to 2 hours since ingestion. In the tablet/food group, only 12 of 73 patients (16%) presented within 1 hour since ingestion (recommended by the 1997 position statement), and 3 patients presented >12 hours since ingestion. In the soluble/fluid phase group, only 9 of 50 patients (18%) presented within 1 hour since ingestion, and 2 patients presented >12 hours since ingestion. The reticular/empty phase group contained the greatest number of patients presenting within 2 to 4 hours since ingestion, and 3 patients presented within 1 hour since ingestion. There was no association between the time that had elapsed since ingestion and the amount of drug ingested in any of the groups (Figure 4).

**FIGURE 5:**
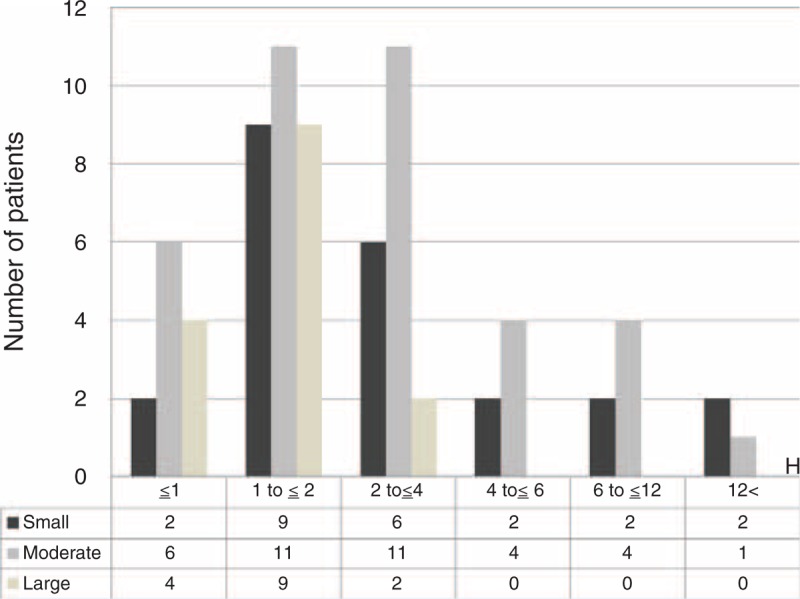
Correlation between time elapsed and amount of gastric contents.

Figure 5 shows that most patients in the tablet/food phase group had presented within 1 to 2 hours of ingestion.

In this study, there was also no significant association between the time since ingestion and the types of drugs ingested. The residual stomach contents before the performance of gastric lavage on admission of patients with oral drug overdoses were variable.

## DISCUSSION

Gastric lavage has not been considered routine for the initial management of intoxicated patients since the publication of guidelines on the indications for gastric lavage. The 1997 position statement states that gastric lavage may be considered for potentially life-threatening overdoses only if it can be performed within 1 hour. However, this recommendation was based on experimental studies of retrieval efficiency in animals,^9,10^ human volunteers,^11^ and a clinical study.^12^ On the contrary, the efficiency of retrieval was variable in another clinical study. Comstock et al^13^ reported that the lavage recovery varied from 6% to 33% with an overall rate of 14%. These studies emphasized the retrieval efficiency. However, the patient's phase of gastric emptying before the performance of gastric lavage is often unknown.

In this study, we demonstrated that the patients in each digestive phase group had a normal histogram. Among the 3 groups, the number of patients seen within 1 to 2 hours since ingestion was higher in the tablet/food and soluble/fluid phase groups (27 of 73 and 18 of 50 patients, respectively). Only 12 of 73 patients (16%) in the tablet/food phase of digestion presented within 1 hour of ingestion. It has been thought that the residual stomach contents in patients who have overdosed are likely to remain beyond 1 hour. Three patients in the tablet/food group and 2 in the soluble/fluid phase group presented >12 hours since ingestion. Three patients in the reticular/empty phase group presented within 1 hour since ingestion. The residual content before gastric lavage was variable among the patients, which may have influenced the efficiency of retrieval of residual toxic substances from the stomach. This study demonstrated no association between the type of oral drug and the time that had elapsed since ingestion. Furthermore, in the clinical setting, it is thought that patients take different amounts and combinations of drugs and meals at various times. The absorption of tablets can easily be affected by several factors, such as gastric pH and variations in gastric secretions.^14^ In addition, intoxication is likely to be associated with hypomotility and a marked delay in gastric emptying, which can influence the clinical course.^15^

This study may provide another clinical observation of the efficacy of gastric lavage. When gastric lavage is performed, the tube is usually inserted through the fornix and body of the stomach with the patient lying on the left side.^16^ However, the tube is not always placed precisely within the residual substance.^17^ Successful retrieval of stomach contents may be difficult in the solid phase of digestion.^18^ In our study, for patients with gastric contents in the tablet/food phase, the residual content was located in the fornix in 32 patients, in the fornix and body in 22 patients, and in the fornix, body, and antrum in 19 patients. Thus, some residual content was located in the fornix in all of these patients. Furthermore, in 3 patients who had presented >12 hours after ingestion, the residual content was located in the fornix. This means that assessment of the distribution of residual content may be influenced not only by positioning but also by the form of the stomach. This may make it difficult to place the stomach tube in the optimal position for irrigation. These factors may have affected the efficacy of gastric lavage.

Historically, there has been concern that complications associated with gastric lavage might outweigh the possible benefits to patients. However, when a patient becomes intoxicated with high-risk drugs, it may be difficult to determine whether the drug contents in the stomach should be retrieved or not. The decision about when or whether to remove the stomach contents should be made based on the risk assessment of the particular intoxication and the benefits and potential risks of gastric lavage for each patient.^19^ The toxicity of the residual substances in the stomach might be underestimated. We are sure that gastric lavage should not be performed routinely; however, the time that has elapsed since oral ingestion should not be overly emphasized. This study has shown that residual stomach contents may be present >1 hour after ingestion. Thus, the time that has elapsed since ingestion should not be a priority consideration when determining whether to perform gastric lavage. We must carefully consider the toxicity of residual substances and the risk–benefit relationship for gastric lavage. In this study, we used endoscopy. Gastric lavage does not always result in successful retrieval of residual gastric contents. Use of endoscopy might contribute to more successful retrieval of residual stomach contents.^20,21^ The residual gastric contents in overdosed patients on admission vary according to the ingested dose, time since ingestion, and various other factors. This may influence the efficiency of gastric lavage for retrieval of residual toxic substances from the stomach. This study may contribute to the development of a strategy for treatment of patients who have orally overdosed on drugs.

### Limitations

Our analysis has several potential limitations. The conclusions are limited by a classification of patients that depended on endoscopic assessment of the stomach contents. We did not evaluate other digestive tract areas, including the duodenum. In addition, a careful history regarding emesis and meal ingestion was not obtained.

## CONCLUSIONS

The residual stomach contents on admission of patients with oral drug overdoses before the performance of gastric lavage were variable. This may influence the efficiency of gastric lavage for retrieval of residual toxic substances from the stomach. This study may contribute to the development of a strategy for treating patients who have orally overdosed on drugs in the future.
